# *N*-[2-(Acetamido)­eth­yl]-2-hy­droxy­benzamide

**DOI:** 10.1107/S1600536811003680

**Published:** 2011-02-05

**Authors:** Michał Kozłowski, Wanda Radecka-Paryzek, Maciej Kubicki

**Affiliations:** a Department of Chemistry, Adam Mickiewicz University, Grunwaldzka 6, 60-780 Poznań, Poland

## Abstract

In the title mol­ecule, C_11_H_14_N_2_O_3_, an intra­molecular O—H⋯O hydrogen bond closes an almost planar [maximum deviation = 0.022 (13) Å] six-membered ring and enforces the *cis* conformation of the keto group with respect to the hy­droxy substituent. In the crystal, inter­molecular N—H⋯O hydrogen bonds link the moleclues into ribbons extended along [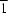
10]. Weak inter­molecular C—H⋯O inter­actions further consolidate the crystal packing.

## Related literature

For general background to ribonucleic acid, see: Franklin (2001 ▶); Komiyama *et al.* (1999 ▶); Kuzuya *et al.* (2006 ▶); Morrow & Iranzo (2004 ▶); Nüttymäki & Lönnberg (2006 ▶). Some crystal structures of similar mol­ecules have been reported, for instance *N*-salicyloylglycine (Smeets *et al.*, 1985 ▶), 2-(*N*-(2-(2-hy­droxy­benzamido)­ethyl­ammonio­eth­yl)amino­carbon­yl) phen­ol­ate (Liu *et al.*, 2006 ▶) and *N*-(2-Amino­eth­yl)-2-hy­droxy­benzamide picrate (Yu *et al.*, 2003 ▶). More crystal structures of analogs can be found in Cambridge Structural Database (Allen, 2002 ▶).
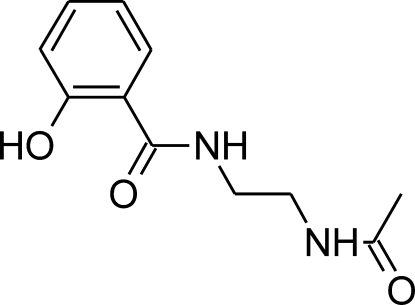


## Experimental

### 

#### Crystal data

C_11_H_14_N_2_O_3_
*M* *_r_* = 222.24
Monoclinic, 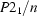

*a* = 8.642 (3) Å
*b* = 4.9702 (18) Å
*c* = 24.972 (3) Å
β = 95.14 (2)°
*V* = 1068.3 (6) Å^3^
*Z* = 4
Mo *K*α radiation
μ = 0.10 mm^−1^
*T* = 90 K
0.3 × 0.2 × 0.15 mm

#### Data collection

Oxford Diffraction Xcalibur Eos diffractometer
Absorption correction: multi-scan (*CrysAlis PRO*; Oxford Diffraction, 2009 ▶) *T*_min_ = 0.111, *T*_max_ = 1.000
4347 measured reflections
2439 independent reflections
1504 reflections with *I* > 2σ(*I*)
*R*_int_ = 0.039

#### Refinement

*R*[*F* ^2^ > 2σ(*F* ^2^)] = 0.058
*wR*(*F* ^2^) = 0.162
*S* = 1.04
2439 reflections
158 parameters
H atoms treated by a mixture of independent and constrained refinement
Δρ_max_ = 0.29 e Å^−3^
Δρ_min_ = −0.34 e Å^−3^

### 

Data collection: *CrysAlis PRO* (Oxford Diffraction, 2009 ▶); cell refinement: *CrysAlis PRO*; data reduction: *CrysAlis PRO*; program(s) used to solve structure: *SIR92* (Altomare *et al.*, 1993 ▶); program(s) used to refine structure: *SHELXL97* (Sheldrick, 2008 ▶); molecular graphics: *SHELXTL* (Sheldrick, 2008 ▶); software used to prepare material for publication: *SHELXL97*.

## Supplementary Material

Crystal structure: contains datablocks I, global. DOI: 10.1107/S1600536811003680/cv5045sup1.cif

Structure factors: contains datablocks I. DOI: 10.1107/S1600536811003680/cv5045Isup2.hkl

Additional supplementary materials:  crystallographic information; 3D view; checkCIF report

## Figures and Tables

**Table 1:** Hydrogen-bond geometry (Å, °)

*D*—H⋯*A*	*D*—H	H⋯*A*	*D*⋯*A*	*D*—H⋯*A*
O1—H1⋯O7	1.11 (4)	1.51 (4)	2.534 (3)	150 (3)
N8—H8⋯O12^i^	0.91 (3)	2.02 (3)	2.895 (3)	160 (2)
N11—H11⋯O7^ii^	0.89 (3)	2.16 (3)	3.040 (3)	174 (2)
C3—H3⋯O12^i^	0.95	2.47	3.404 (4)	169
C6—H6⋯O1^iii^	0.95	2.47	3.325 (4)	150

Symmetry codes: (i) 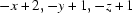; (ii) 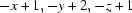; (iii) 
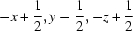
.

**Fig. 1.**
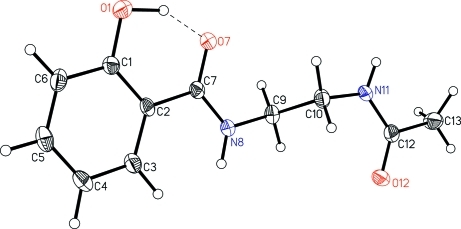
View of I showing the atomic numbering and 50% probability displacement ellipsoids. Hydrogen atoms are depicted as spheres with arbitrary radii. Intramolecular hydrogen bond is shown as dashed line.

**Fig. 2.**
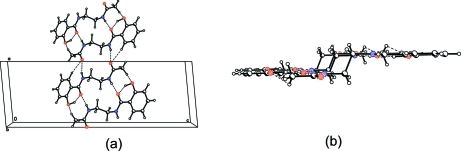
Two views: (a) face-on and (b) side-on of the hydrogen-bonded chain of molecules of I. Hydrogen bonds are shown as dashed lines.

**Fig. 3.**
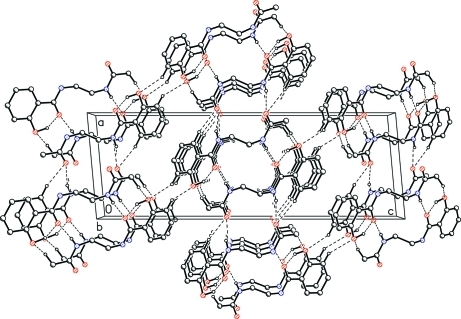
A portion of the crystal packing as seen approximately along b-direction. Hydrogen bonds are shown as dashed lines.

## supplementary crystallographic information

### Comment

Ribonucleic acid, which mediates genetic information encoded in DNA, is one of the most important compounds in life. If only one RNA can be chosen from many RNAs in cells and selectively cleaved at desired site, it opens the way to new RNA science (*e.g.* regulation of expression of a specific gene, advanced therapy, RNA manipulation) (Kuzuya *et al.*, 2006). During the past decade, mimics for RNA-cleaving enzymes, ribonucleases, have received special attention (Nüttymäki *et al.*, 2006). The first artificial nucleases capable of cleaving RNA oligonucleotides in a selective manner were DNA conjugates of lanthanide(III) ion complexes (Komiyama *et al.*, 1999; Franklin, 2001; Morrow *et al.*, 2004). The title compound (I, Scheme 1) was isolated during efforts to prepare new synthetic ribonuclease precursors as part of our research program involving the study of the nonselective and selective hydrolysis of RNA by lanthanide complexes.

The conformation of the CNCCNCC chain in (I) is *tg^+^tg^-^t* (*t* - *trans*, *g* - *gauche*), as can be seen from the values of the torsion angles. Intramolecular hydrogen bond between hydroxy group and O7 oxygen atom causes closing of the six-membered nearly planar (within 0.022 (13) Å) ring (Fig. 1). This bond is strong and causes the changes in the geometry of involved fragments: lengthening of both O—H (1.11 (4) Å) and C=O (1.255 (3) Å) bonds. This ring is almost coplanar with the phenyl ring plane, the dihedral angle between the two planes is 1.6 (9)°. In the Cambridge Structural Database (Allen, 2002; Version 5.31 of Nov. 2009, updated August 2010) there are 229 fragments of 2-hydroxy-N-monosubstituted- benzamide, and both O—H···O and N—H···O (with hydroxy group as an acceptor) are almost equally represented in the sample. Of course, the different hydrogen bond schemes are connected with the different C1—C2—C7—N8 torsion angles, which are close to 180° for the former and close to 0° for the latter possibility (*cf.* Fig. 2). The overall conformation of the molecule can be described as two almost planar (within 0.022 (2) Å) and nearly parallel (the dihedral angle is 5.65 (16)°) fragments C1···C9 and C10···C13.

In the crystal structure, the variety of hydrogen bonds connects the molecules of I into the hydrogen-bonded chains of molecules (*cf.* Table 1). The pairs of almost linear N11—H11···O7(*1 - x,2 - y,1 - z*) and N8—H8···O12(*2 - x,1 - y,1 - z*) hydrogen bonds join the molecules in centrosymmetric dimers, the graph set connected with these interactions are *R*^2^_2_(14). Each of these bonds is accompanied by secondary however still relatively short and directional C—H···O interactions (Table 1). As can be seen in Fig. 3 these bonds in general join two different "storeys" of the molecules in alternating manner. Therefore these interactions create the double ribbons of molecules which expand approximately along [-110] direction. The interactions between these motifs are only very week.

### Experimental

To a solution of ethylenediamine (0,3972 g, 2 mmol) in THF (7 ml) *O*-acetylsalicyloyl chloride (0,268 ml, 4 mmol) in THF (7 ml) was added dropwise with stirring. The reaction was carried out for 24 h in ambient temperature. The reaction mixture was evaporated to dryness and purified by silica gel column chromatography by elution with CH_2_Cl_2_/methanol (98:2). Crystals suitable for X-ray diffraction analysis were formed by slow evaporation from CH_2_Cl_2_/methanol (1:1) after one week.

ESI-MS m/*z* (%) = 221 (100 {C_11_H_13_N_2_O_3_^-^}); 245 (100 {C_11_H_14_N_2_O_3_+Na^+^}).

Elemental analysis calculated for C_11_H_14_N_2_O_3_: C, 59.45; H, 6.35; N, 12.60; found C, 58.95; H, 6.00; N, 12.20.

^1^H-NMR p.p.m.: 12.46 (*s*); 7.90 (*s*); 7.48 (*d*); 7.38 (*t*); 6.98 (*d*); 6.89 (*t*); 6.10 (*s*); 3.56 (*t*); 2.05 (*s*).

### Refinement

C-bound H atoms were geometrically positioned (C—H 0.95-0.99 Å) and refined as riding, with Uiso(H) = 1.2-1.5 Ueq(C). The rest H atoms were found in the diffrence Fourier maps and isotropically refined.

### Crystal data

**Table:**

C_11_H_14_N_2_O_3_	*F*(000) = 472
*M**_r_* = 222.24	*D*_x_ = 1.38 Mg m^−^^3^
Monoclinic, *P*2_1_/*n*	Mo *K*α radiation, λ = 0.71073 Å
Hall symbol: -P 2yn	Cell parameters from 990 reflections
*a* = 8.642 (3) Å	θ = 3.0–29.0°
*b* = 4.9702 (18) Å	µ = 0.10 mm^−^^1^
*c* = 24.972 (3) Å	*T* = 90 K
β = 95.14 (2)°	Block, yellow
*V* = 1068.3 (6) Å^3^	0.3 × 0.2 × 0.15 mm
*Z* = 4	

### Data collection

**Table:**

Oxford Diffraction Xcalibur Eos diffractometer	2439 independent reflections
Radiation source: Enhance (Mo) X-ray Source	1504 reflections with *I* > 2σ(*I*)
graphite	*R*_int_ = 0.039
Detector resolution: 16.1544 pixels mm^-1^	θ_max_ = 29.2°, θ_min_ = 3.3°
ω scans	*h* = −11→6
Absorption correction: multi-scan (*CrysAlis PRO*; Oxford Diffraction, 2009)	*k* = −4→6
*T*_min_ = 0.111, *T*_max_ = 1.000	*l* = −28→33
4347 measured reflections	

### Refinement

**Table:**

Refinement on *F*^2^	Primary atom site location: structure-invariant direct methods
Least-squares matrix: full	Secondary atom site location: difference Fourier map
*R*[*F*^2^ > 2σ(*F*^2^)] = 0.058	Hydrogen site location: inferred from neighbouring sites
*wR*(*F*^2^) = 0.162	H atoms treated by a mixture of independent and constrained refinement
*S* = 1.04	*w* = 1/[σ^2^(*F*_o_^2^) + (0.0581*P*)^2^ + 0.0266*P*] where *P* = (*F*_o_^2^ + 2*F*_c_^2^)/3
2439 reflections	(Δ/σ)_max_ < 0.001
158 parameters	Δρ_max_ = 0.29 e Å^−^^3^
0 restraints	Δρ_min_ = −0.34 e Å^−^^3^

### Special details

**Table:**

Geometry. All e.s.d.'s (except the e.s.d. in the dihedral angle between two l.s. planes) are estimated using the full covariance matrix. The cell e.s.d.'s are taken into account individually in the estimation of e.s.d.'s in distances, angles and torsion angles; correlations between e.s.d.'s in cell parameters are only used when they are defined by crystal symmetry. An approximate (isotropic) treatment of cell e.s.d.'s is used for estimating e.s.d.'s involving l.s. planes.
Refinement. Refinement of *F*^2^ against ALL reflections. The weighted *R*-factor *wR* and goodness of fit *S* are based on *F*^2^, conventional *R*-factors *R* are based on *F*, with *F* set to zero for negative *F*^2^. The threshold expression of *F*^2^ > σ(*F*^2^) is used only for calculating *R*-factors(gt) *etc*. and is not relevant to the choice of reflections for refinement. *R*-factors based on *F*^2^ are statistically about twice as large as those based on *F*, and *R*- factors based on ALL data will be even larger.

### Fractional atomic coordinates and isotropic or equivalent isotropic displacement parameters (Å^2^)

**Table:**

	*x*	*y*	*z*	*U*_iso_*/*U*_eq_	
C1	0.4433 (3)	0.3212 (5)	0.31898 (9)	0.0195 (5)	
O1	0.3176 (2)	0.4713 (3)	0.32788 (7)	0.0273 (5)	
H1	0.357 (4)	0.597 (7)	0.3630 (15)	0.078 (12)*	
C2	0.5847 (3)	0.3520 (4)	0.34941 (9)	0.0163 (5)	
C3	0.7073 (3)	0.1857 (5)	0.33760 (10)	0.0211 (6)	
H3	0.8049	0.2046	0.3579	0.025*	
C4	0.6913 (3)	−0.0051 (5)	0.29741 (10)	0.0237 (6)	
H4	0.7763	−0.1170	0.2904	0.028*	
C5	0.5507 (3)	−0.0306 (5)	0.26766 (10)	0.0239 (6)	
H5	0.5385	−0.1613	0.2398	0.029*	
C6	0.4275 (3)	0.1306 (5)	0.27763 (10)	0.0233 (6)	
H6	0.3313	0.1127	0.2564	0.028*	
C7	0.5993 (3)	0.5583 (4)	0.39258 (9)	0.0165 (5)	
O7	0.48629 (18)	0.7039 (3)	0.40218 (6)	0.0215 (4)	
N8	0.7353 (2)	0.5851 (4)	0.42095 (8)	0.0191 (5)	
H8	0.815 (3)	0.471 (5)	0.4157 (10)	0.030 (8)*	
C9	0.7594 (3)	0.7863 (5)	0.46326 (10)	0.0204 (5)	
H9A	0.7091	0.9568	0.4508	0.024*	
H9B	0.8722	0.8209	0.4706	0.024*	
C10	0.6929 (3)	0.6963 (5)	0.51550 (9)	0.0214 (6)	
H10A	0.5782	0.6873	0.5095	0.026*	
H10B	0.7316	0.5136	0.5251	0.026*	
N11	0.7358 (2)	0.8778 (4)	0.55967 (8)	0.0212 (5)	
H11	0.666 (3)	0.991 (5)	0.5708 (11)	0.028 (7)*	
C12	0.8726 (3)	0.8603 (5)	0.58797 (10)	0.0208 (6)	
O12	0.97079 (19)	0.6959 (3)	0.57683 (7)	0.0249 (4)	
C13	0.8981 (3)	1.0561 (5)	0.63381 (11)	0.0296 (6)	
H13A	0.9890	1.0007	0.6575	0.044*	
H13B	0.8062	1.0589	0.6541	0.044*	
H13C	0.9157	1.2363	0.6196	0.044*	

### Atomic displacement parameters (Å^2^)

**Table:**

	*U*^11^	*U*^22^	*U*^33^	*U*^12^	*U*^13^	*U*^23^
C1	0.0211 (12)	0.0222 (13)	0.0154 (12)	0.0028 (10)	0.0029 (10)	0.0010 (10)
O1	0.0243 (10)	0.0317 (10)	0.0247 (10)	0.0087 (8)	−0.0043 (8)	−0.0064 (9)
C2	0.0225 (12)	0.0153 (11)	0.0117 (11)	0.0012 (10)	0.0051 (10)	0.0011 (10)
C3	0.0221 (13)	0.0245 (13)	0.0172 (13)	−0.0004 (10)	0.0038 (10)	−0.0019 (11)
C4	0.0285 (14)	0.0230 (13)	0.0213 (13)	0.0025 (11)	0.0110 (11)	−0.0018 (11)
C5	0.0318 (15)	0.0245 (13)	0.0164 (13)	−0.0070 (12)	0.0081 (11)	−0.0029 (11)
C6	0.0243 (13)	0.0309 (14)	0.0142 (12)	−0.0034 (11)	−0.0005 (10)	0.0009 (11)
C7	0.0206 (12)	0.0162 (12)	0.0130 (12)	0.0003 (10)	0.0043 (9)	0.0038 (10)
O7	0.0210 (9)	0.0265 (9)	0.0170 (9)	0.0046 (7)	0.0022 (7)	−0.0016 (7)
N8	0.0200 (11)	0.0226 (11)	0.0148 (11)	0.0031 (9)	0.0014 (8)	−0.0037 (9)
C9	0.0245 (13)	0.0208 (12)	0.0158 (12)	−0.0018 (10)	0.0015 (10)	−0.0015 (11)
C10	0.0220 (12)	0.0261 (13)	0.0164 (13)	−0.0029 (11)	0.0024 (10)	−0.0027 (11)
N11	0.0184 (11)	0.0301 (12)	0.0152 (11)	0.0051 (9)	0.0019 (9)	−0.0060 (9)
C12	0.0217 (13)	0.0242 (13)	0.0168 (13)	−0.0007 (11)	0.0036 (10)	0.0011 (11)
O12	0.0212 (9)	0.0308 (10)	0.0228 (10)	0.0061 (8)	0.0021 (7)	−0.0068 (8)
C13	0.0240 (14)	0.0373 (15)	0.0262 (15)	0.0070 (12)	−0.0051 (11)	−0.0109 (13)

### Geometric parameters (Å, °)

**Table:**

C1—O1	1.352 (3)	N8—C9	1.456 (3)
C1—C2	1.389 (3)	N8—H8	0.91 (3)
C1—C6	1.399 (3)	C9—C10	1.538 (4)
O1—H1	1.11 (4)	C9—H9A	0.9900
C2—C3	1.395 (3)	C9—H9B	0.9900
C2—C7	1.485 (3)	C10—N11	1.447 (3)
C3—C4	1.379 (3)	C10—H10A	0.9900
C3—H3	0.9500	C10—H10B	0.9900
C4—C5	1.372 (4)	N11—C12	1.325 (3)
C4—H4	0.9500	N11—H11	0.89 (3)
C5—C6	1.373 (3)	C12—O12	1.228 (3)
C5—H5	0.9500	C12—C13	1.504 (3)
C6—H6	0.9500	C13—H13A	0.9800
C7—O7	1.255 (3)	C13—H13B	0.9800
C7—N8	1.323 (3)	C13—H13C	0.9800
			
O1—C1—C2	122.0 (2)	N8—C9—C10	112.0 (2)
O1—C1—C6	117.9 (2)	N8—C9—H9A	109.2
C2—C1—C6	120.1 (2)	C10—C9—H9A	109.2
C1—O1—H1	104.2 (18)	N8—C9—H9B	109.2
C1—C2—C3	117.8 (2)	C10—C9—H9B	109.2
C1—C2—C7	119.1 (2)	H9A—C9—H9B	107.9
C3—C2—C7	123.1 (2)	N11—C10—C9	112.1 (2)
C4—C3—C2	122.2 (2)	N11—C10—H10A	109.2
C4—C3—H3	118.9	C9—C10—H10A	109.2
C2—C3—H3	118.9	N11—C10—H10B	109.2
C5—C4—C3	118.9 (2)	C9—C10—H10B	109.2
C5—C4—H4	120.5	H10A—C10—H10B	107.9
C3—C4—H4	120.5	C12—N11—C10	121.4 (2)
C4—C5—C6	120.8 (2)	C12—N11—H11	118.2 (17)
C4—C5—H5	119.6	C10—N11—H11	120.0 (18)
C6—C5—H5	119.6	O12—C12—N11	121.6 (2)
C5—C6—C1	120.2 (2)	O12—C12—C13	123.1 (2)
C5—C6—H6	119.9	N11—C12—C13	115.2 (2)
C1—C6—H6	119.9	C12—C13—H13A	109.5
O7—C7—N8	120.6 (2)	C12—C13—H13B	109.5
O7—C7—C2	121.3 (2)	H13A—C13—H13B	109.5
N8—C7—C2	118.1 (2)	C12—C13—H13C	109.5
C7—N8—C9	121.4 (2)	H13A—C13—H13C	109.5
C7—N8—H8	120.5 (16)	H13B—C13—H13C	109.5
C9—N8—H8	118.1 (17)		
			
O1—C1—C2—C3	179.3 (2)	C1—C2—C7—O7	0.3 (3)
C6—C1—C2—C3	−0.8 (3)	C3—C2—C7—O7	−179.8 (2)
O1—C1—C2—C7	−0.8 (3)	C1—C2—C7—N8	−179.7 (2)
C6—C1—C2—C7	179.1 (2)	C3—C2—C7—N8	0.2 (3)
C1—C2—C3—C4	−0.2 (3)	O7—C7—N8—C9	−1.2 (3)
C7—C2—C3—C4	179.9 (2)	C2—C7—N8—C9	178.81 (19)
C2—C3—C4—C5	0.7 (4)	C7—N8—C9—C10	78.9 (3)
C3—C4—C5—C6	−0.1 (4)	N8—C9—C10—N11	171.87 (19)
C4—C5—C6—C1	−0.9 (4)	C9—C10—N11—C12	−82.2 (3)
O1—C1—C6—C5	−178.7 (2)	C10—N11—C12—O12	2.5 (4)
C2—C1—C6—C5	1.4 (4)	C10—N11—C12—C13	−177.9 (2)

### Hydrogen-bond geometry (Å, °)

**Table:**

*D*—H···*A*	*D*—H	H···*A*	*D*···*A*	*D*—H···*A*
O1—H1···O7	1.11 (4)	1.51 (4)	2.534 (3)	150 (3)
N8—H8···O12^i^	0.91 (3)	2.02 (3)	2.895 (3)	160 (2)
N11—H11···O7^ii^	0.89 (3)	2.16 (3)	3.040 (3)	174 (2)
C3—H3···O12^i^	0.95	2.47	3.404 (4)	169.
C6—H6···O1^iii^	0.95	2.47	3.325 (4)	150.

Symmetry codes: (i) −*x*+2, −*y*+1, −*z*+1; (ii) −*x*+1, −*y*+2, −*z*+1; (iii) −*x*+1/2, *y*−1/2, −*z*+1/2.
